# Decreased Expression of the FOXO3a Gene Is Associated with Poor Prognosis in Primary Gastric Adenocarcinoma Patients

**DOI:** 10.1371/journal.pone.0078158

**Published:** 2013-10-23

**Authors:** Xiao-bo Yang, Jing-jing Zhao, Chun-yu Huang, Qi-jing Wang, Ke Pan, Dan-dan Wang, Qiu-zhong Pan, Shan-shan Jiang, Lin Lv, Xiang Gao, Huang-wei Chen, Jia-yin Yao, Min Zhi, Jian-chuan Xia

**Affiliations:** 1 State Key Laboratory of Oncology in Southern China and Department of Experimental Research, Sun Yat-sen University Cancer Center, Guangzhou, P.R. China; 2 Department of Gastroenterology, the Sixth Affiliated Hospital, Sun Yat-sen University, Guangzhou, P.R. China; 3 Department of Biotherapy Center, Sun Yat-sen University Cancer Center, Guangzhou, P.R. China; 4 Department of Endoscopy, Sun Yat-sen University Cancer Center, Guangzhou, P.R. China; The University of Hong Kong, China

## Abstract

**Background:**

FOXO3a, a member of the forkhead class ‘O’ (FOXO) transcription factor family, controls a wide spectrum of biological processes, such as DNA damage repair, apoptosis, and cell cycle regulation. FOXO3a has been shown to be a tumor suppressor in various cancers. This study investigated the expression of FOXO3a in primary gastric adenocarcinomas and its prognostic value for primary gastric adenocarcinoma patients.

**Methods:**

Real-time quantitative RT-PCR (qRT-PCR), western blotting, and immunohistochemical staining were used to detect FOXO3a expression in primary gastric cancerous surgical specimens and adjacent non-tumorous tissues.

**Results:**

Our data showed that the expression of FOXO3a mRNA (p = 0.03) and protein (p = 0.019) was lower in cancerous tissues compared with their adjacent non-tumorous tissues. In addition, the chi-square test revealed that low FOXO3a expression was significantly correlated with larger tumor size (p = 0.007), poor histopathological classification (p = 0.029), depth of invasion (p = 0.049), local lymph node metastasis (p = 0.013), distant metastasis (p = 0.013) and AJCC staging (p<0.001). Kaplan-Meier survival analysis demonstrated that low expression of FOXO3a was significantly correlated with a poor prognosis for gastric cancer patients (p<0.001). The multivariate analysis showed that FOXO3a expression was an independent prognostic factor of the overall survival rate of patients with primary gastric adenocarcinoma.

**Conclusion:**

Our study suggested that decreased FOXO3a expression may play an important role in the progression of gastric cancer. FOXO3a could be a valuable prognostic marker as well as a potential molecular therapy target for gastric cancer patients.

## Introduction

Gastric carcinoma is one of the leading causes of cancer mortality in the world, with an estimated one million new cases every year [1]–[4]. An increasing number of new cases of gastric cancer have been diagnosed recently, particularly in East Asian countries, such as China, Japan and Korea, as well as in other developing countries [4]. Despite great advancements in diagnosis and treatment modalities for this disease, especially surgery, chemotherapy, and radiotherapy, its survival rate remains very low [5]. To improve patient outcome, it is clinically important to find efficient new targets for the early diagnosis and effective treatment of gastric carcinoma. Gastric carcinogenesis is a multifactorial and multistep process that involves the activation of oncogenes and inactivation of tumor suppressor genes at different stages of gastric cancer progression [6], [7]. Several new oncogenes and tumor suppressor genes associated with gastric cancer have been identified that may be helpful for early diagnosis and for the development of targeted therapies [6], [7]. However, clarifying additional molecular markers and investigating their molecular mechanisms that are involved in gastric cancer are critical for improved diagnosis and treatment of gastric cancer [8]–[11].

FOXO (Forkhead box, class ‘O’) comprises a subgroup of the winged helix or forkhead transcription factors that regulate a wide range of biological functions, including development, growth, stress resistance, apoptosis, cell cycle, immunity, metabolism, and aging [12], [13]. FOXOs promote tumor suppression by the induction of proteins that mediate cell cycle arrest, apoptosis, and DNA damage repair. In humans, four members of the FOXO transcription factors have been identified: FOXO1, FOXO3a, FOXO4 and FOXO6 [14], [15]; they share a high degree of evolutionary conservation, especially in their forkhead DNA-binding domains [16]–[18]. FOXO3a is localized in the nucleus, where it activates or represses the transcription of target genes [15]. Upon stimulation by growth factors, FOXO3a is phosphorylated and accelerates the nuclear exclusion of FOXO3a, thereby inhibiting its ability to bind to DNA [15]. Previous studies showed that FOXO3a is a suppressor of primary tumor growth and is negatively regulated by growth factors [19]–[23]. During tumor development, inhibition of the transcriptional activity of FOXO3a promotes cell transformation, tumor progression, and angiogenesis [24]–[27]. In addition, FOXO3a overexpression has been shown to inhibit breast tumor growth and decrease tumor size [27],[28]. Furthermore, the abnormal expression of FOXO3a correlates with poor survival for breast cancer patients [27]. These results indicate that FOXO3a plays a tumor suppressor role.

However, to our knowledge, few reports have been published concerning the role of FOXO3a in gastric cancer. The expression and the prognostic value of FOXO3a in human primary gastric cancers have not yet been assessed. Thus, in the present study, we analyzed the FOXO3a expression level in gastric cancers using real-time quantitative RT-PCR (qRT-PCR), western blotting and immunohistochemical analysis. Furthermore, we identified the relationship between FOXO3a expression and the clinicopathological features of gastric cancer. The prognostic value of FOXO3a for the post-resection survival of gastric cancer patients was also evaluated.

## Results

### FOXO3a mRNA expression analyzed by qRT-PCR

**Figure 1:**
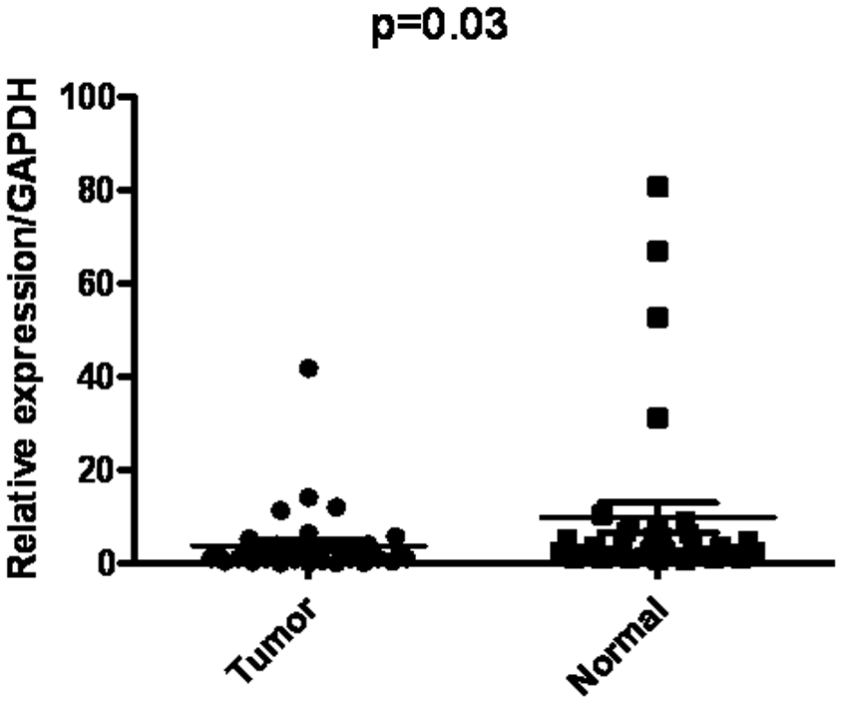
Real-time quantitative RT-PCR analysis of FOXO3a expression in gastric cancer surgical specimens. The relative mRNA expression of FOXO3a was significantly lower in 24 of 35 (68.6%) gastric cancer tissues compared with the corresponding non-tumorous tissues. (FOXO3a/GAPDH, n = 35, p = 0.03).

The mRNA level of FOXO3a was measured by qRT-PCR in 35 paired cancerous and adjacent non-gastric cancer tissues from primary gastric cancer patients. The FOXO3a mRNA expression level was significantly lower in 24 of 35 (68.6%) gastric cancer tissues compared with the corresponding non-tumorous tissues (p = 0.03), as shown in Figure 1.

### FOXO3a expression analyzed by Western Blotting

**Figure 2:**
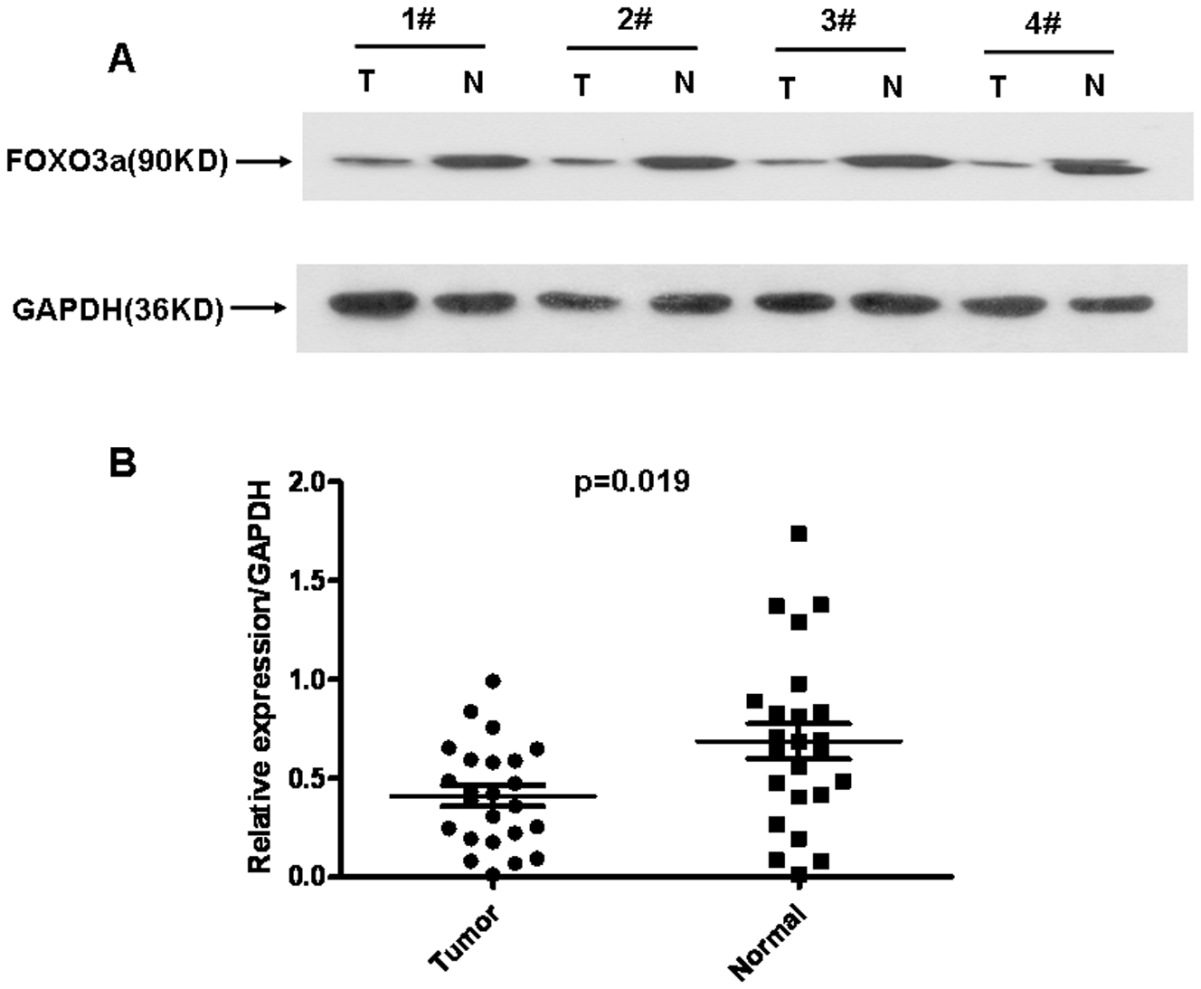
Protein expression of FOXO3a in gastric cancer as evaluated by western blotting. (**A**) Representative result of FOXO3a protein expression in 4 paired gastric cancerous and the matched adjacent non-tumorous tissues (T, gastric cancer tissues; N, matched non-tumorous gastric mucosa). (**B**) Relative FOXO3a protein expression levels was remarkably decreased in 18 of 24 (75%) gastric tumor tissues compared with the corresponding adjacent non-tumorous tissues, (FOXO3a/GAPDH, n = 24, p = 0.019).

Western blotting was performed on 24 gastric cancerous tissues as well as the matched non-tumorous tissues to evaluate FOXO3a protein expression. The results showed a band for FOXO3a at 90 kDa, and the protein expression intensity of FOXO3a was measured by analyzing the protein bands using densitometry software. We found that FOXO3a expression was remarkably decreased in 18 of 24 (75%) gastric tumor tissues compared with the corresponding adjacent non-tumorous tissues (p = 0.019, Figure 2), which was consistent with the qRT-PCR results.

### Immunohistochemical analysis of FOXO3a expression in gastric cancer clinical samples and its relationship with clinicopathological parameters

**Figure 3:**
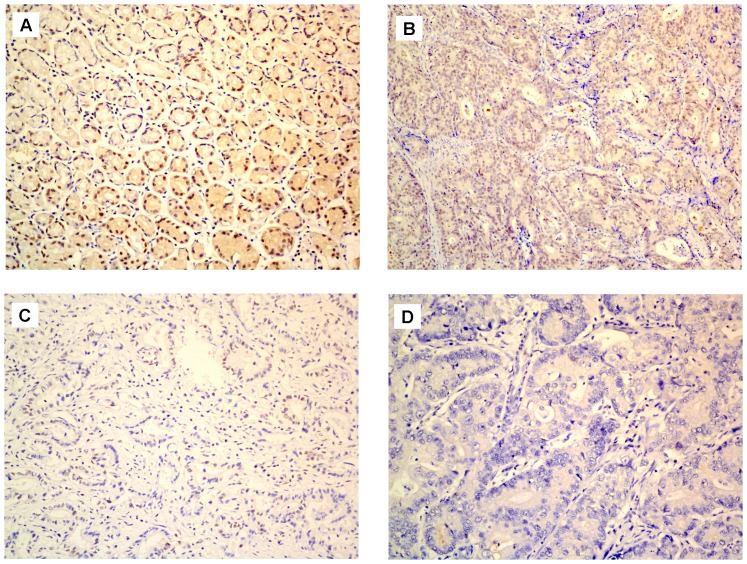
FOXO3a protein expression in gastric cancer and surrounding non-tumorous tissues detected by immunohistochemistry. (**A**) Normal gastric tissues distant from the tumor were scored as FOXO3a (+++); (**B**) Well differentiated gastric cancer was scored as FOXO3a (++); (**C**) Moderately differentiated gastric cancer was scored as FOXO3a (+); (**D**) Poorly differentiated gastric cancer was scored as FOXO3a (-). Original magnification: 200×.

Immunohistochemical analysis was performed on the 174 gastric cancer tissue paraffin sections to further investigate the FOXO3a expression in situ and its relationship with clinicopathological parameters. We found that FOXO3a was expressed at various levels in the gastric tumor tissues and the adjacent non-tumorous tissue samples (Figure 3). Among the 174 gastric cancer samples, 94 cases showed high FOXO3a expression (FOXO3a ++ or FOXO3a +++), whereas the remaining 80 cases displayed low FOXO3a expression (FOXO3a - or FOXO3a +) (Figure 3, Table 1). The adjacent non-tumorous tissues showed the strongest FOXO3a positive staining (Figure 3A).

**Table 1:** Correlation between FOXO3a expression and clinicopathological variables of 174 gastric cancer cases.

Clinicopathologic variables	number of each group	FOXO3a expression		*p* value
		low	high	
All cases	174	80	94	
Age(years)				0.478
<60	95	46	49	
≧60	79	34	45	
Gender				0.165
Male	124	53	71	
Female	50	27	23	
Tumor size(cm)				0.007^a^
<4	71	24	47	
≧4	103	56	47	
Histologic grade				0.029^a^
well	3	1	2	
moderate	58	20	38	
poor	113	59	54	
Depth of invasion				0.049^a^
T1–T2	40	13	27	
T3–T4	134	67	67	
Lymph node metastasis				0.013^a^
N0	46	12	32	
N1–N3	128	66	62	
Distance metastasis				0.013^a^
no	158	68	90	
yes	16	12	4	
AJCC staging				<0.001^a^
I–II	69	20	49	
III–IV	105	60	45	

a *p* value<0.05.

The Chi square analysis showed that the expression level of FOXO3a in tumor tissues was significantly correlated with various clinicopathological parameters, such as tumor size (p = 0.007), histological grade (p = 0.029), depth of invasion (p = 0.049), local lymph node metastasis (p = 0.013), distance metastasis (p = 0.013) and AJCC staging (p<0.001), but not with age (p = 0.478) or gender (p = 0.165) (Table 1).

### The relationship of FOXO3a expression and patient survival

**Figure 4:**
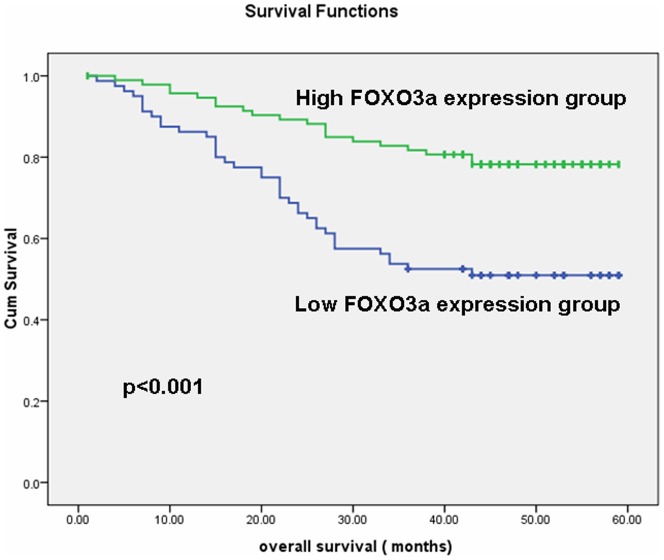
The Kaplan-Meier survival curves of gastric cancer patients (n = 174) after surgical resection. The gastric cancer patients were divided into low FOXO3a expression (FOXO3a- or FOXO3a+) and high FOXO3a expression (FOXO3a++ or FOXO3a+++) groups. Patients in the low FOXO3a expression group showed significantly poorer survival than those in the high FOXO3a expression group (log-rank test: p<0.001).

The prognostic value of FOXO3a in gastric cancer patients was evaluated by survival analysis of the high and low FOXO3a expression groups. The 5-year overall survival rates in patients with low and high FOXO3a expression in their tumor tissue samples were 51.3% and 72.4%, respectively. The patients with high FOXO3a expression had a significantly better overall survival than those with low expression (p<0.001, log-rank test, Figure 4). Univariate Cox regression analysis indicated that FOXO3a expression, depth of invasion, local lymph node metastasis and distant metastasis were significantly associated with the overall survival of gastric cancer patients (Table 2). Multivariate Cox regression analyses showed that FOXO3a expression can be used as an independent predictor for overall survival of gastric cancer patients (Table 2).

**Table 2:** Univariate and multivariate analysis of overall survival of gastric cancer patients.

Variables	Univariate analysis			Multivariate analysis		
	HR	95% CI	*p* value	HR	95% CI	*p* value
FOXO3a expression (lowvhigh)	0.355	0.207–0.609	<0.001^a^	0.469	0.270–0.815	0.007^a^
Age (<60v≧60)	1.079	0.647–1.799	0.770			
Gender (malevfemale)	0.846	0.486–1.472	0.553			
Tumor size (<4cmv≧4cm)	1.667	0.957–2.902	0.071			
Depth of invasion(T1–T2vT3–T4)	6.766	2.117–21.631	0.001^a^	5.829	1.805–18.826	0.003^a^
Lymph node metastasis(N0vN1–N4)	3.736	1.605–8.695	0.002^a^	2.182	0.925–5.150	0.075
Distance metastasis(novyes)	4.546	2.400–8.612	<0.001^a^	3.695	1.922–7.101	<0.001^a^
Histologic grade(well/moderate/poor)	1.453	0.864–2.442	0.090			

*HR* Hazard ratio, *CI* confidence interval.

a *p* value<0.05.

## Discussion

Tumor progression depends on factors that are intrinsic to tumor cells, including growth factors and their cognate receptors, extracellular matrix proteins, proteases, chemokines, and cellular adhesion molecules. The expression of these factors is influenced by the environment and the microenvironment of the tumor, as well as by genetic and epigenetic factors [29]. Members of the FOXO family of transcription factors have been implicated in tumorigenesis [30]. FOXO3a, which is one of the FOXO transcription factors, has been shown to function as a tumor suppressor in both ERα-positive and ERα-negative breast cancers [19], [31]. In the present study, we evaluated the expression of FOXO3a and its prognostic role in human primary gastric cancer with a relatively large series of clinical tissue samples for the first time.

Similar to previous studies on breast and prostate cancer [32], [33], we found, using qRT-PCR and western blotting analysis, that FOXO3a expression was decreased at the mRNA and protein levels, respectively, in most tumor tissues compared to their adjacent non-tumorous tissues. Immunohistochemical staining in situ analysis also showed that FOXO3a expression was low in the tumor tissues of approximately half of the selected post-resection primary cancer patients. Furthermore, a decreased expression of FOXO3a was significantly associated with larger tumor size, poorly differentiated adenocarcinoma, lymph node metastasis and distant metastasis, suggesting that abnormal FOXO3a expression might be involved in gastric cancer tumor progression and metastasis and that FOXO3a could also play a tumor suppressor role in gastric cancer.

Further retrospective survival analysis of 174 post-resection gastric cancer patients revealed that low FOXO3a expression was significantly correlated with shorter survival time of gastric adenocarcinoma patients. Furthermore, the univariate and multivariate analyses demonstrated that FOXO3a expression was an independent predictor of overall survival (OS) in gastric adenocarcinoma patients. These results suggest that FOXO3a may serve as a valuable prognostic biomarker for gastric cancer patients after surgery. These results are consistent with the findings of Habashy HO et al., who reported that nuclear localization of FOXO3a and its subsequent transcriptional activity are a marker of good prognosis among breast cancer patients [32]. Thus, FOXO3a might play an important role in various types of cancer.

In conclusion, FOXO3a was found for the first time to have decreased expression in gastric cancer, and thus, it may play a tumor suppressor role in gastric cancer. FOXO3a expression might serve as a valuable prognostic biomarker for gastric cancer patients. Our results suggest that FOXO3a has the potential to be used as a target for therapeutic interventions for gastric cancer patients.

## Materials and Methods

### Ethics statement

The research was approved by the Ethics Committee of Sun Yat-sen University Cancer Center, and written informed consent was obtained from each patient involved in the study.

### Patients and tumor tissue samples

From January 2003 to December 2006, clinicopathological data from 174 gastric cancer patients who underwent surgical resection at Sun Yat-sen University Cancer Center were retrospectively analyzed. Patients who met the following eligibility criteria were included: (1) diagnosis of gastric adenocarcinoma identified by histopathological examination; (2) surgical history that included gastrectomy plus lymphadenectomy (limited or extended); (3) availability of complete follow-up data; (4) no preoperative treatment, such as chemotherapy or radiotherapy; (5) no history of familial malignancy or other synchronous malignancy (such as GIST, esophageal cancer, or colorectal cancer); (6) no recurrent gastric cancer or remnant gastric cancer; and (7) survival through the perioperative period. The tumor resections and D2 lymphadenectomies were performed by experienced surgeons, and the surgical procedures, which followed the Japanese Gastric Cancer Association (JGCA) guidelines, were similar in all patients who underwent radical resections.

Fresh gastric cancer and adjacent non-tumorous tissue samples (n = 35) were obtained from 35 gastric cancer patients who underwent surgical resection at the Sun Yat-sen University Cancer Center between 2010 and 2011. After surgical resection, the fresh tissue samples were immediately immersed in RNAlater (Ambion Inc., USA) and stored at 4°C overnight to allow thorough penetration of the tissues; the samples were then frozen at −80°C until RNA and protein extraction were performed. Both the tumor tissues and the adjacent non-tumorous tissues, which were located more than 2 cm away from the gastric cancer, were sampled and then verified by pathological examination.

Paraffin-embedded samples were obtained from 174 gastric cancer patients who underwent surgical resection at the Sun Yat-sen University Cancer Center between 2003 and 2006. The follow-up data from the gastric cancer patients in this study were available and complete. The postoperative follow-up occurred at our outpatient department and included clinical and laboratory examinations every 3 months for the first 2 years, every 6 months during the third to fifth years, and annually for an additional 5 years or until patient death, whichever occurred first. Overall survival, which was defined as the time from the operation to the time of patient death or the last follow-up, was used as a measure of prognosis. Clinical and pathologic classification and staging were determined according to the American Joint Committee on Cancer (AJCC) TNM staging system. The clinical information related to the 174 gastric cancer was described in Table 3.

**Table 3:** Clinical characteristics and FOXO3a expression of 174 patient samples of gastric cancer.

Characteristics	Number of cases (%)
**Age(years)**	
<60	95 (54.6)
≧60	79 (45.4)
**Gender**	
Male	124 (71.3)
Female	50 (28.7)
**Tumor size(cm)**	
≧4	71 (40.8)
≧4	103 (59.2)
**Histologic grade**	
well	3 (1.7)
moderate	58 (33.3)
poor	113 (65.0)
**T classification**	
T1–T2	40 (23.0)
T3–T4	134 (77.0)
**N classification**	
N0	46 (26.4)
N1–N3	128 (73.6)
**M classification**	
M0	158 (90.8)
M1	16(9.2)
**Clinical stage**	
I–II	69(39.6)
III–IV	105 (60.4)
**Vital status (at follow-up)**	
Alive	115(66.1)
Death	59 (33.9)
**Expression of FOXO3a**	
Low expression	80(46.0)
High expression	94(54.0)

### RNA extraction and real-time quantitative RT-PCR

Total RNA was extracted using the TRIzol solution (Invitrogen, USA) according to the manufacturer's instructions. RNase-free DNase I was used to remove DNA contamination. The total RNA concentration and quantity were assessed by absorbency at 260 nm using a Nanodrop spectrophotometer (ND-1000, Thermo Scientific, USA). First-strand cDNA synthesis was performed using 2 µg of total RNA and M-MLV reverse transcriptase, according to the manufacturer's instructions (Promega, USA). The resulting cDNAs were subjected to real-time quantitative RT-PCR analysis to evaluate the relative expression levels of FOXO3a and GAPDH (an internal control) using the following primers: 5′-GCAAGCACAGAGTTGGATGA-3′ (F) and 5′-CAGGTCGTCCATGAGGTTTT -3′(R) for FOXO3a; and 5′-CTCCTCCTGTTCGACAGTCAGC-3′ (F) and 5′- CCCAATACGACCAAATCCGTT-3′(R)for GAPDH. Each 15 µl reaction volume contained 0.5 µl of cDNA that was synthesized as described above, 7.5 µl of 2×SYBR Green master mix (Invitrogen, USA), and 200 nM of each pair of oligonucleotide primers described above. The cycling parameters began with an initial step of 95°C for 10 minutes, followed by 45 cycles of 90°C for 30 seconds and 60°C for 60 seconds; then, a melting curve analysis was performed. The Ct was measured during the exponential amplification phase, and the amplification plots were analyzed using the software provided with the instrument (SDS 2.3). The relative expression levels of the target gene were normalized to that of the internal control gene, GAPDH. The data were analyzed using the comparative threshold cycle (2^−ΔΔCT^) method.

### Protein extraction and western blotting analysis

The frozen gastric cancer samples, including the tumor tissues and non-tumorous control tissues, were homogenized in a RIPA lysis buffer, and the lysates were cleared by centrifugation (14,000 rpm) at 4°C for 30 minutes. Protein samples of approximately 40 µg were run on 12% SDS-PAGE gels and transferred to PVDF membranes. After blocking the non-specific binding sites for 60 minutes with 5% non-fat milk, the membranes were incubated with primary monoclonal antibodies against FOXO3a (Epitomics, USA, at a 1∶600 dilution) or GAPDH (Medical & Biological Laboratories, Japan, at a 1∶10000 dilution) overnight at 4°C. Next, the membranes were subjected to three 15-minute washes with TBST and then incubated with HRP-conjugated secondary antibody (at a 1∶2000 dilution) for 45 minutes at room temperature. The membranes were washed three more times with TBST and developed using an enhanced chemiluminescence system (ECL, Cell Signaling Technologies).

### Immunohistochemistry

Paraffin-embedded tissue blocks were sectioned for immunohistochemistry. The sections were deparaffinized and rehydrated with graded ethanol. For antigen retrieval, the slides were immersed in EDTA (1 mmol/L, pH 8.0) and boiled for 15 minutes in a microwave oven. After rinsing with PBS, the endogenous peroxidase was blocked with 0.3% hydrogen peroxide for 15 minutes at room temperature. The slides were incubated with the primary antibody (mouse anti- FOXO3a monoclonal antibody, Epitomics, USA, at a 1∶500 dilution) overnight in a humidified chamber at 4°C. The sections were washed three times with PBS, incubated with horseradish peroxidase-conjugated secondary antibody (Envision™ Detection Kit, GK500705, Gene Tech) at 37°C for 30 minutes, and then washed three more times with PBS. Finally, 3, 3′-diaminobenzidine tetrahydrochloride (DAB) was used for signal development, and the sections were counterstained with 20% hematoxylin. The slides were dehydrated, cleared and evaluated. Each sample was incubated with an isotypic antibody dilution under the same experimental conditions as the negative control.

### Semi-quantitative method

The total FOXO3a immunostaining score was calculated as both the percentage of positively stained tumor cells and the staining intensity. The percent positivity was scored as “0” (<5%, negative), “1” (5%–25%, sporadic), “2” (25%–50%, focal), or “3” (>50%, diffuse). The staining intensity was scored as “0” (no staining), “1” (weakly stained), “2” (moderately stained), or “3” (strongly stained). Both the percentage of positive cells and the staining intensity were evaluated under double-blind conditions. The FOXO3a immunostaining score was calculated as the percentage positive score × the staining intensity score and ranged from 0 to 9. We defined the FOXO3a expression levels as follows: ‘−’ (score 0–1), ‘+’ (score 2–3), ‘++’ (score 4–6) and ‘+++’ (score >6). Based on the FOXO3a expression levels, the gastric cancer patients were divided into two groups: the low FOXO3a expression group (FOXO3a - or FOXO3a +) and the high FOXO3a expression group (FOXO3a ++ or FOXO3a +++).

### Statistical analysis

A paired-sample t-test was used to compare the FOXO3a mRNA levels in the tumor tissue samples with their adjacent non-tumorous tissue samples. The χ2 test for proportion was used to analyze the relationship between the FOXO3a expression level and various clinicopathological characteristics. The overall survival curves were calculated with the Kaplan-Meier method and were analyzed with the log-rank test. The Cox proportional-hazard analysis was used for univariate and multivariate analyses to explore the effect of the clinicopathological variables and FOXO3a expression on survival. Only the factors which were found to have statistically significant associations with overall survival based on a univariate analysis would be included in a multivariate Cox proportional hazards model to adjust for the effects of the covariates. Furthermore, variables that were highly associated with others were excluded from the final multivariate Cox proportional hazards model. Proportional hazards assumption was tested by adding a timedependent version of all variables in the model. For categorization purposes, FOXO3a expression was entered into the model in two different groups, where “low expression” group was compared to “high expression”group. In the same way, age was entered into the model in two different groups, where “<60” group was compared to “≧60”group. Gender was entered into the model in two different groups, where “male”group was compared to “female” group. Tumor size was entered into the model in two different groups, where “<4” was compared to “≧4”. Depth of invasion was entered into the model in two different groups, where “T1–T2” group was compared to “T3–T4” group. Lymph node metastasis was entered into the model in two different groups, where “N0” group was compared to “N1–N4” group. Distance metastasis was entered into the model in two different groups, where “no” group was compared to “yes” group. Histological grade was entered into the model in three different groups, where “well” and “moderate” group were compared to “poor” group. A two-sided p-value <0.05 was considered to be statistically significant. All statistical analyses were performed with the SPSS software (version 17.0; SPSS Inc., Chicago, IL, USA).
